# The Effect of Family Functioning on Depressive Symptoms in Adolescents: A Moderated Mediation Model

**DOI:** 10.1155/da/9965173

**Published:** 2026-02-01

**Authors:** Zhang Jiayuan, Zhang Hui, Li Yang, Zhou Yuqiu

**Affiliations:** ^1^ Department of Psychological Nursing, Harbin Medical University, Daqing, Heilongjiang Province, China, hrbmu.edu.cn; ^2^ Key Laboratory of Basic Research and Health Management on Chronic Diseases in Heilongjiang Province, Daqing, China

**Keywords:** adolescents, depressive symptoms, family functioning, insecure attachment, sensory processing sensitivity

## Abstract

**Background:**

Family dysfunction and insecure attachment are established risk factors for adolescent depressive symptoms, yet individual differences in sensory processing may influence vulnerability to these environmental stressors.

**Objective:**

To explore the mediating role of insecure attachment and the moderating effect of sensory processing sensitivity (SPS) in the relationship between family functioning and adolescent depressive symptoms.

**Methods:**

This study employed a cross‐sectional design, with 503 adolescents recruited via convenience sampling in October 2023. Participants completed self‐report questionnaires assessing family functioning, insecure attachment, SPS, and depressive symptoms. SPSS 26.0 was used to conduct moderated mediation analyses to examine the complex interactions among these variables.

**Results:**

The results showed that family functioning was directly associated with adolescent depressive symptoms and also was indirectly associated with depressive symptoms through insecure attachment. Additionally, SPS was found to statistically moderate both the direct and indirect pathways. Specifically, the negative association between poor family functioning and depressive symptoms and the statistical mediating pathway through insecure attachment were more pronounced in adolescents with higher SPS.

**Conclusion:**

Impaired family functioning and insecure attachment were associated with higher levels of depressive symptoms in adolescents. SPS appeared to strengthen these associations, highlighting the importance of considering individual differences in sensory sensitivity when addressing adolescent mental health. Tailoring interventions to strengthen family support and attachment security, especially for adolescents with heightened SPS, may help mitigate the risk of depressive symptoms. This study emphasizes the need for family‐centered interventions to foster resilience against adolescent depressive symptoms.

## 1. Introduction

According to the Global Issues in Adolescent Health released by WHO, suicide caused by depression has become the second leading cause of death among adolescents worldwide [1]. The Diagnostic and Statistical Manual of Mental Disorders, Fifth Edition (DSM‐5) defines depression as a spectrum of mood disorders, which includes major depressive disorder, persistent depressive disorder, depressive symptoms due to other medical conditions, as well as other specified depressive disorders and unspecified depressive disorders, all characterized by the presence of depressive episodes [2]. Adolescence is a critical period for individual physical and psychological development [3]. Previous research has indicated a significant increase in the risk of depressive symptoms upon entering adolescence [4]. A recent meta‐analysis revealed that approximately one‐fifth of children and adolescents worldwide suffer from depressive symptoms or exhibit depressive symptoms, with this proportion increasing over time [5]. Depressive symptoms is considered the primary mental health concern for adolescents due to its high incidence and risk, and is closely associated with adverse health outcomes such as substance abuse, self‐injury, suicidal behaviors, and other crisis events [6]. Despite the availability of treatment, current interventions for depressive disorders often remain only partially effective, underscoring the urgent need to identify potential contributing factors. Understanding these factors is critical for improving prevention strategies and clinical interventions, as they hold significant implications for adolescent mental health.

The family, as the most direct and important microsystem, has a significant impact on the physical and mental development of children and adolescents [7]. Family dysfunction is a crucial factor influencing the mental health of adolescents [8]. Family functioning is considered to be a comprehensive assessment of the effectiveness of family members in managing emotional connections, family communication, internal rules, and external events [9]. Shek [10] further refined family functioning into three core components: mutual relationships, communication and adaptation, and conflict and harmony. Recent empirical evidence has consistently demonstrated the association between family functioning and adolescent mental health [11–13]. This relationship is particularly salient in adolescent depressive symptoms. For instance, higher scores in family adaptability are associated with lower levels of depressive symptoms [14], while children from conflicted and violent families exhibit higher levels of depressive symptoms [15]. Although previous studies have extensively discussed the relationship between family functioning or specific aspects of it and adolescent depressive symptoms, there is less emphasis on the role of individual characteristics in this relationship, which warrants further exploration. The sensory processing sensitivity (SPS) model suggests that individuals with “susceptible” characteristics are more likely to experience psychological and behavioral problems such as depressive symptoms in negative environments [16]. Additionally, attachment theory highlights the importance of early emotional bonds between caregivers and children, with insecure attachment often leading to difficulties in coping with stress and emotional regulation during adolescence [17]. Attachment plays a crucial role in shaping how adolescents respond to family dynamics [18], and its interaction with family functioning may significantly influence the development of depressive symptoms.

To understand the complex interplay between these variables, this study adopts an integrated theoretical framework drawing from Bronfenbrenner’s ecological systems theory and the developmental psychopathology perspective. According to Bronfenbrenner’s ecological framework, family functioning represents the most proximal microsystem influencing adolescent development through daily interactions and emotional climate [19]. The developmental psychopathology framework further emphasizes that attachment serves as a critical mechanism through which family experiences become internalized into working models that guide emotional regulation [20]. Poor family functioning disrupts secure attachment development, leading to maladaptive internal working models that increase vulnerability to depressive symptoms. Integrating these perspectives with differential susceptibility theory, we propose that the strength of these pathways varies as a function of SPS. This integrated framework explains how family‐level functioning, individual‐level attachment patterns, and temperamental sensitivities converge to shape adolescent depressive symptoms risk, moving beyond simple additive models to articulate specific mechanisms and boundary conditions.

### 1.1. The Mediating Role of Insecure Attachment

The concept of family functioning encompasses the support provided by the family in individual socialization, development of various abilities, and establishment of a nurturing material and spiritual environment for their physiological and psychological growth to meet the developmental needs of each family member [21]. According to the system model of family functioning theory, the execution of family functioning is closely intertwined with family relationships [22]. A positive and harmonious family atmosphere contributes to adolescents forming secure attachment relationships with their parents, enhancing their emotional well‐being, and reducing the likelihood of experiencing depressive symptoms. Numerous studies have indicated that inadequate family functioning, such as parental conflicts and unhealthy couple relationships, can result in diminished quality of parent–child interaction, leading to a loss of safety and intimacy within parent–child relationships among children and adolescents, ultimately giving rise to insecure parent–child attachments [23, 24].

The attachment theory posits that the quality of attachment between children and their parents can significantly impact their internal working models, influencing how they perceive and evaluate themselves and others. Secure and stable attachment relationships foster feelings of self‐worth and trust in others, while insecure attachments can lead to negative psychological representations of self and others, as well as a range of negative emotional, cognitive, and behavioral patterns that contribute to depressive symptoms [25, 26]. Multiple meta‐analyses have demonstrated that insecure attachment is a risk factor for childhood depressive symptoms [27–29]. A study on the impact of family environment on adolescent depressive symptoms explored the roles of attachment and psychological resilience in the relationship between the two, and found that insecure attachment partially mediated the relationship between adverse family environment and adolescent depressive symptoms [30]. Therefore, both family functioning and insecure attachment play crucial roles in adolescent depressive symptoms. Poor family functioning may directly contribute to insecure attachment, thereby increasing the risk of adolescent depressive symptoms.

### 1.2. The Moderating Role of SPS

The family system theory hold the opinion that children’s emotional problems are influenced by both familial and individual factors [31]. While a series of studies have confirmed the predictive role of poor family functioning and insecure attachment on adolescent depressive symptoms, there exist individual differences in the level of depressive symptoms among adolescents with different characteristics when exposed to adverse environments.

The model of children’s developmental environmental sensitivity emphasizes that the interaction between different environmental sensitivities and impacts leads to diverse development outcomes [32]. SPS, as a trait with dual environmental susceptibility, reflects individual differences in sensitivity to environmental stimuli at the temperament level [33]. Studies have shown that compared with low SPS children, children with high SPS tend to have stronger reactions to environmental stimuli and deeper processing, and are more susceptible to the influence of the external environment [16, 34]. Therefore, children with different SPS levels have different reactivity to the environment, resulting in different effects of environmental factors on them [35, 36]. SPS is an indicator of temperament sensitivity, and children with high SPS are more susceptible to the influence of the family environment. Slagt et al. [34] examined the changes in parenting styles and the impact of SPS on children’s externalization behavior. The study found that when parental negative parenting decreased, the externalization behavior of high SPS children decreased more than that of low SPS children; when parental negative parenting increased, the externalization problems of high SPS children increased more [34]. Previous studies have shown that SPS is significantly positively correlated with adolescent depressive symptoms, and deeper cognitive processing and higher emotional responses are believed to be core features of individuals with high environmental sensitivity [37].

According to differential susceptibility theory, individuals with high SPS represent a “for better and for worse” phenotype, exhibiting enhanced susceptibility to both positive and negative environmental influences [38]. This dual‐sensitivity characteristic suggests that the impact of insecure attachment on depressive symptoms may be particularly pronounced among adolescents with high SPS. When experiencing insecure attachment, adolescents with high SPS may demonstrate heightened emotional reactivity and deeper cognitive processing of negative relational experiences, thereby amplifying the risk of depressive symptoms. Emerging empirical evidence supports this moderating role. Study demonstrated that children with high SPS showed stronger associations between attachment security and subsequent emotional adjustment [39], while Bosmans et al. [40] found that the combination of insecure attachment and high SPS predicted higher levels of internalizing problems among adolescents.

Furthermore, recent studies have explored the relationship between SPS and attachment patterns. One scoping review found that SPS and attachment patterns are linked [41]. Additionally, research has highlighted the interactive effects of SPS and internal working models of attachment in emotion regulation. Specifically, higher SPS was found to be associated with positive attachment representations [39]. This suggests that attachment plays a crucial role in shaping how children with high SPS respond to their emotional environments, further underscoring the importance of considering both factors in understanding adolescent development. It is important to note that while SPS may moderate the relationship between insecure attachment and depressive symptoms, its role becomes particularly relevant when considering how individuals process and respond to their attachment experiences. Adolescents with high SPS may ruminate more deeply on negative attachment‐related cognition and emotions, leading to a stronger translation of attachment insecurity into depressive symptoms.

### 1.3. The Current Study

Previous research has shown that family functioning, insecure attachment, and SPS all play significant roles in adolescent depressive symptoms. However, the specific relationships between these variables remain underexplored. Therefore, the current study aims to fill this gap by investigating the interplay between family functioning, insecure attachment, and SPS, and exploring how these factors collectively influence adolescent depressive symptoms. Based on the environmental sensitivity theory and attachment theory, this study examines the mediating role of insecure attachment and the moderating role of SPS in the relationship between family functioning and adolescent depressive symptoms. Specifically, the current study proposes three hypotheses.

**Hypothesis 1.**

Family functioning is directly associated with adolescent depressive symptoms, such that higher levels of family functioning are associated with lower levels of depressive symptoms.

**Hypothesis 2.**

Insecure attachment mediates the relationship between family functioning and adolescent depressive symptoms, such that family functioning impacts adolescent depressive symptoms through its influence on insecure attachment.

**Hypothesis 3.**

SPS moderates the relationship between family functioning and adolescent depressive symptoms. Specifically, SPS may moderate the latter half of the mediation pathway, influencing the link between insecure attachment and adolescent depressive symptoms.

**Figure 1:**
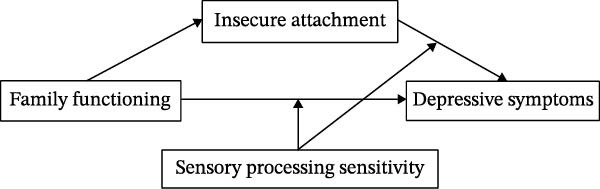
Research hypothesis.

Based on these hypotheses, the study constructs a moderated mediation model, as illustrated in Figure 1. By exploring these mechanisms, the study aims to deepen our understanding of the complex interactions between individual sensitivities and family dynamics, offering insights into potential targets for intervention in adolescent depressive symptoms.

## 2. Materials and Methods

### 2.1. Study Design

This study adopted a cross‐sectional survey design to examine the relationships among family functioning, insecure attachment, SPS, and depressive symptoms in adolescents. A cross‐sectional design was selected for two primary reasons. First, due to practical constraints related to school schedules, limited access periods, and the need to minimize disruption to classroom instruction. Second, the present study aimed to provide an initial test of the theoretically proposed moderated mediation model in a natural school setting, serving as a foundational step for subsequent longitudinal follow‐up research. Although cross‐sectional data restrict causal interpretations, such designs are considered appropriate for mechanism‐exploration studies that examine associations among psychological constructs within a theoretical framework. Additionally, given that the sample included adolescents from both urban and rural areas, the cross‐sectional approach allowed for the identification of contextual heterogeneity in psychological development. The study design, therefore, integrates a theory‐driven analytical framework with practical considerations of school‐based data collection, providing meaningful preliminary evidence while laying the groundwork for more rigorous longitudinal investigations.

### 2.2. Participants and Recruitment

In October 2023, a total of 503 middle school students from two public middle schools in Yichun City, Heilongjiang Province, participated in the study. The sample size was determined based on recommendations from prior mediation and moderation research [42], which suggest that approximately 400–500 participants are adequate for detecting medium effect sizes in moderated mediation models with statistical power ≥0.80. The selection process involved a convenience sampling method, where schools were chosen based on accessibility, and students were invited to participate. Initial contact with the schools was established through outreach to school administrators, who were provided with detailed information about the study’s purpose, methodology, and ethical considerations. After obtaining approval from the schools, students were informed about the study and asked to voluntarily participate.

The participants had a mean age of 15.60 years (SD = 0.89), with 255 boys and 248 girls. Of the total sample, 208 participants (41.3%) were from rural areas, and 295 (58.7%) were from urban areas. The sample was further categorized by grade level, consisting of 190 students from Grade One (surveyed in four classes), 173 from Grade Two (surveyed in four classes), and 140 from Grade Three (surveyed in three classes).

### 2.3. Measures

#### 2.3.1. Family Assessment Device (FAD)

FAD was developed by Epstein in 1983 and consists of seven dimensions: problem solving, communication, roles, emotional response, affective involvement, behavior control, and overall functioning. Epstein suggested that the general functioning (GF) subscale can be used as a standalone tool for assessing family functioning and has been widely utilized [43]. In this study, the Chinese version of GF subscale of FAD was employed to assess the family functioning (GF) of adolescents [44]. The scale comprises 12 items (for example: “Each family member feels understood and recognized for who they truly are by others.” And “In times of crisis, our family members can rely on each other for support.”) and utilizes a four‐point scoring system ranging from 1 to 4, with 1 indicating “completely inconsistent” and 4 indicating “completely consistent.” The range of scores was from 12 to 48. A higher score indicates better family functioning. The Cronbach’s *α* coefficient for this measurement was found to be 0.924.

#### 2.3.2. Adolescent Attachment Questionnaire (AAQ)

The AAQ compiled by West et al. [45] in 1998 was used to measure the insecure attachment of adolescents. The AAQ is a self‐report scale consisting of nine items (for example: “My parent is always disappointing me,” “I’m afraid that I will lose my parent’s love”), scored on a five‐point Likert scale. The total score ranges between 9 and 45. The higher the score, the higher the level of insecure attachment of adolescents. The Chinese version of the AAQ has good reliability and validity [46]. The Cronbach’s alpha of AAQ yielded good consistency in this study (*α* = .912).

#### 2.3.3. Highly Sensitive Child Scale (HSC)

The HSC was initially developed by Pluess et al. [47] in 2018 and subsequently revised by Liu Qianwen [48] into the Chinese version. This scale comprises nine items (for example: “Staying in noisy places makes me feel uncomfortable,” “I don’t like certain things in my life to change”). The scale adopted a 7‐point scoring system (“1” = “very unlike me” and “7” = “very like me”) with the range scores of 7–49. The higher the score, the higher the level of SPS. The Cronbach’s *α* coefficient of the scale in this study was 0.921.

#### 2.3.4. Center for Epidemiological Studies Depression Scale (CES‐D)

The CES‐D was developed by Radloff [49] and revised by Chen Zhiyan and Xinying [50] into Chinese version which included 20 questions (for example: “I get upset over small things,” “I don’t feel like eating much; my appetite is poor”) and was scored on a 0–3 scale (“0” means “occasionally or not” and “3” means “most of the time or continuously”), with higher scores indicating higher levels of depressive symptoms. Previous studies have confirmed that the scale has good reliability and validity, and the Cronbach’s *α* coefficient of the scale in this study was 0.974.

#### 2.3.5. Measurement Model Validation

To assess the validity of the measurement model, a confirmatory factor analysis (CFA) was conducted with all four latent constructs (family functioning, insecure attachment, SPS, and depressive symptoms) included simultaneously. The results indicated an acceptable model fit: *χ* ^2^/df = 1.141, RMSEA = 0.017, CFI = 0.990, TLI = 0.990, GFI = 0.906, and IFI = 0.990. In addition, the convergent validity and discriminant validity were tested. It is observed in Table 1. If the value of the average variance variance (AVE) is greater than 0.5 and the value of the combined reliability (CR) is greater than 0.7, it indicates that the variable has good convergent validity [51]. It is observed in appendix Table A1 (reliability and validity test).

**Table 1:** Discriminant validity test.

Constructs	Family functioning	Insecure attachment	Sensory processing sensitivity	Depressive symptom
Family functioning	0.709	—	—	—
Insecure attachment	−0.410 ^∗∗^	0.733	—	—
Sensory processing sensitivity	−0.393 ^∗∗^	0.273 ^∗∗^	0.755	—
Depressive symptom	−0.384 ^∗∗^	0.334 ^∗∗^	0.156 ^∗∗^	0.808

*Note:* ^∗∗^ indicates *p*  < 0.01, and the diagonal is the square root of the AVE mean variance variance draw.

### 2.4. Procedure

The study received approval from the university’s Research Ethics Committee. Before the investigation, participants were informed that their responses would be kept strictly confidential. After obtaining informed consent from students and their guardians, the group test was conducted in a classroom setting. The questionnaires were administered in paper format to ensure a structured response process. To ensure the quality of the data collected, training sessions were held with the staff prior to the research. The staff were instructed on how to distribute the questionnaires and follow the unified guidance language for explaining the instructions to the respondents. The distribution and collection of the questionnaires were managed by two graduate students. They were responsible for explaining the guidelines and requirements to each class, ensuring that all students understood the process. Once completed, the questionnaires were collected on‐site and checked for completeness and clarity. Any incomplete or unclear responses were flagged immediately to ensure the integrity of the data. Additionally, the graduate students monitored the process to ensure that the questionnaires were completed accurately and returned promptly.

### 2.5. Statistical Analysis

The study used SPSS 26.0 and the SPSS macro PROCESS compiled by Hayes (2013) for model testing. Prior to the main analyses, key assumptions for regression were verified. Multicollinearity was assessed using variance inflation factor (VIF) values. Normality of residuals was confirmed through Kolmogorov–Smirnov tests. The analysis procedures were as follows: descriptive statistics and correlation analysis were conducted for all study variables, followed by the application of Model 4 in Hayes’s SPSS PROCESS program to test whether insecure attachment mediates the relationship between family functioning and adolescent depressive symptoms [52]. This step was performed to confirm the existence of a mediation effect. If the mediation effect was significant, Model 15 was then employed to examine the moderate mediation effect, focusing on how SPS moderates the relationship between family functioning, insecure attachment, and depressive symptoms levels. To further examine the moderating effect of SPS, participants were classified into three groups based on their SPS scores using the mean ± 1 SD method: low SPS (scores below M − 1 SD), moderate SPS (scores between M − 1 SD and M + 1 SD), and high SPS (scores above M + 1 SD). The significance of all regression coefficients was tested using the Bootstrap method with 5000 resamples, and indirect effects were considered significant when the 95% bias‐corrected confidence intervals did not include zero.

## 3. Results

### 3.1. Common Bias

To assess the potential impact of common method bias (CMB), Harman’s single‐factor test was conducted. All items from the main study variables were entered into an exploratory factor analysis. The results showed that the first unrotated factor accounted for 32.76% of the total variance, which is below the critical threshold of 40%, indicating that CMB is unlikely to be a major concern in this study.

### 3.2. The Demographic Information of Participants and Their Comparison in Depressive Symptoms

Participants were aged between 14 and 17 years, with a mean age of 15.60 ± 0.89 years. The sample consisted of 255 male participants and 248 female participants. There was no significant gender difference in depressive symptoms between gender (*t* = −0.75, *p* = 0.57). The sample consisted of 208 rural participants and 295 urban participants. A significant gender difference in depressive symptoms was observed, with rural (17.12 ± 6.02) scoring higher than urban(15.61 ± 6.99) (*t* = 2.52, *p*  < 0.01). Regarding grade level, there were 190 participants in the first year (Grade one), 173 participants in the second year (Grade two), and 140 participants in the third year (Grade three). No significant differences in depressive symptoms were observed across grade levels (*F* = 0.998, *p* = 0.370).

### 3.3. Levels of Family Functioning, Insecure Attachment, SPS, and Their Correlations With Depressive Symptoms

The mean score for family functioning was 35.05 ± 5.80, while the mean score for insecure attachment was 20.60 ± 6.39. Additionally, the mean score for SPS was 30.17 ± 7.08, and the mean score for depressive symptoms was 16.24 ± 6.64. Correlation analysis showed that family functioning was significantly negatively correlated with insecure attachment (*r* = −0.38; *p* < 0.01) and depressive symptoms (*r* = −0.36; *p* < 0.01) and SPS (*r* = −0.36; *p* < 0.01). Insecure attachment was significantly positively correlated with depressive symptoms (*r* = 0.31; *p* < 0.01). SPS was significantly positively correlated with depressive symptoms (*r* = 0.15; *p* < 0.01) and insecure attachment (*r* = 0.25; *p* < 0.01).

### 3.4. The Mediation of Insecure Attachment Between Family Functioning and Depressive Symptoms

Using Model 4 of the PROCESS macro, a mediation analysis was conducted while controlling for relevant covariates. The results showed that family functioning was significantly and negatively associated with adolescents’ depressive symptoms (*β* = −0.407, *p*  < 0.001), supporting Hypothesis 1. The model explained 13.8% of the variance in depressive symptoms (*R* ^2^ = 0.138). Family functioning was also significantly and negatively associated with insecure attachment (*β* = −0.420, *p*  < 0.001). This model accounted for 14.8% of the variance in insecure attachment (*R* ^2^ = 0.148). Insecure attachment was positively associated with adolescents’ depressive symptoms (*β* = 0.208, *p*  < 0.001). When family functioning and insecure attachment were entered into the model simultaneously, and with covariates held constant, the negative association between family functioning and depressive symptoms remained significant (*β* = −0.319, *p*  < 0.001). The full mediation model explained 17.3% of the variance in depressive symptoms (*R* ^2^ = 0.173, *f* ^2^ = 0.209) and representing a medium effect size according to Cohen’s guidelines. These findings suggest that insecure attachment statistically accounted for part of the association between family functioning and adolescents’ depressive symptoms, providing support for Hypothesis 2. The results indicated that the mediation effect value of insecure attachment was −0.088, with a 95% confidence interval of [−0.125, −0.055], excluding zero, thereby confirming the significance of the mediation effect. Furthermore, the indirect pathway accounted for 21.62% of the total association, suggesting that 21.62% of the association between family functioning and depressive symptoms operates through insecure attachment. See Table 2 for details.

**Table 2:** The mediation of insecure attachment between family functioning and depressive symptom.

Variable		Value	SE	LLCI	ULCI
Insecure attachment	Total effect	−0.407	0.048	−0.502	−0.312
	Direct effect	−0.319	0.051	−0.419	−0.218
	Indirect effect	−0.088	0.017	−0.125	−0.055

### 3.5. The Moderated Mediation Models

Before testing the moderated mediation model, multicollinearity was examined. The VIF values for all predictors ranged from 1.014 to 1.801, all below the recommended cutoff of 5, indicating no multicollinearity issue.

We tested the moderated mediation model using Model 15, controlling for region, age, grade, gender, with family functioning as the independent variable, depressive symptoms as the dependent variable, insecure attachment as the mediator, and SPS as the moderator to examine the moderated mediation effect.

The results indicated that family functioning was significantly negatively associated with adolescents’ depressive symptoms (*β* = −0.293, *p*  < 0.001). The interaction term between family functioning and SPS also was significantly associated with adolescents’ depressive symptoms (*β* = −0.070, *p*  < 0.001). Furthermore, insecure attachment was significantly positively associated with adolescents’ depressive symptoms (*β* = 0.248, *p*  < 0.001), and the interaction term between insecure attachment and SPS was significantly associated with adolescents’ depressive symptoms (*β* = 0.024, *p*  < 0.001) (Table 3). The overall model explained 22.5% of the variance in depressive symptoms (*R* ^2^ = 0.225).

**Table 3:** The mediate and moderate effect of insecure attachment an sensory processing sensitivity.

Content	*β*	SE	*t*	*p*‐Value	LLCI	ULCI
Constant	16.138	5.739	2.812	<0.001	4.862	27.414
Age	0.708	0.393	0.179	0.857	−0.703	0.844
Grade	−0.255	0.440	−0.580	0.561	−1.122	0.610
Gender	−0.129	0.340	−0.379	0.705	−0.797	0.539
Region	−0.683	0.543	−1.257	0.209	−1.751	0.384
Family functioning	−0.293	0.052	−5.588	<0.001	−0.396	−0.190
Insecure attachment	0.248	0.045	5.447	<0.001	0.158	0.337
Sensory processing sensitivity (SPS)	−0.017	0.041	−0.433	0.664	−0.097	0.062
Family functioning × SPS	−0.070	0.013	−5.126	<0.001	−0.097	−0.043
Insecure attachment × SPS	0.024	0.010	2.309	0.021	0.003	0.043
*R* ^2^	0.225					
*F*	15.916 ^∗∗∗^					

^∗∗^ *p* < 0.001.

^∗∗∗^ *p* < 0.001.

Additionally, the index of moderated mediation (IMM) was calculated to further test the moderated mediation effect. The IMM was −0.010, and its Bootstrap 95% confidence interval [−0.024,−0.003] did not include zero, indicating that the indirect effect of family functioning on depressive symptoms through insecure attachment significantly varied across different levels of SPS.

**Figure 2:**
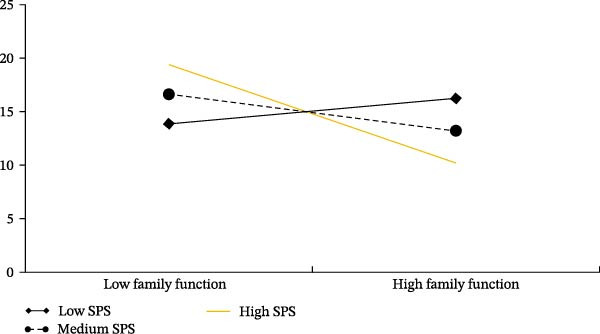
Simple slope plot of the moderating role of sensory processing sensitivity on the relationship between family functioning and depressive symptoms.

To further analyze the moderating role of SPS in the mediation model, a simple slopes analysis was conducted. SPS levels were categorized into high, medium, and low groups using the mean ± 1 standard deviation method. For individuals with low SPS levels, the association between family functioning and depressive symptoms was not significant (*β* = 0.203, *p* = 0.082), with a Bootstrap 95% confidence interval of [−0.026, 0.432]. In contrast, for individuals with medium SPS levels (*β* = −0.293, *p*  < 0.001; Bootstrap 95% confidence interval of [−0.396, −0.190]) and high SPS levels (*β* = −0.791, *p*  < 0.001; Bootstrap 95% confidence interval of [−0.993, −0.587]), the negative association between family functioning and depressive symptoms was significant. Moreover, the strength of the negative association between family functioning and depressive symptoms increased as SPS levels increased (Figure 2).

**Figure 3:**
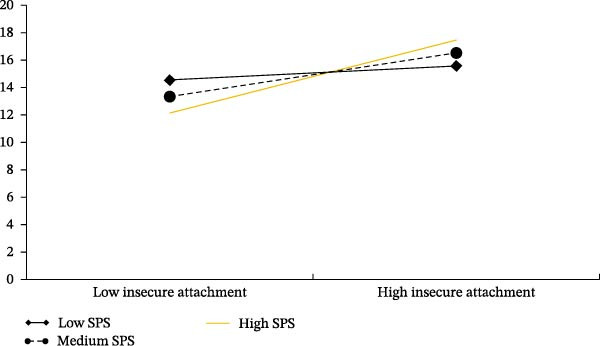
Simple slope plot of the moderating role of sensory processing sensitivity on the relationship between insecure attachment and depressive symptoms.

In the moderating role of SPS in the relationship between insecure attachment and depressive symptoms, for individuals with low SPS levels, the association between insecure attachment and depressive symptoms was not significant (*β* = 0.081, *p* = 0.321), with a Bootstrap 95% confidence interval of [−0.079, 0.241]. However, for individuals with medium SPS levels (*β* = 0.248, *p*  < 0.001; Bootstrap 95% confidence interval of [0.158, 0.337]) and high SPS levels (*β* = 0.415, *p*  < 0.001; Bootstrap 95% confidence interval of [0.240, 0.591]), the positive association between insecure attachment and depressive symptoms was significant. Moreover, the strength of the positive association between insecure attachment and depressive symptoms increased as SPS levels increased (Figure 3).

## 4. Discussion

The current study is grounded in developmental psychopathology and constructs a moderated mediation model to explore the relationships among family functioning, insecure attachment, SPS, and adolescent depressive symptoms. It systematically investigates the mechanisms and influencing factors contributing to adolescent depressive symptoms. The findings indicate that family functioning has a negative association with adolescent depressive symptoms, insecure attachment acts as a mediator between the family environment and adolescent depressive symptoms, and SPS moderates this mediation pathway, aligning with the environmental sensitivity theory. The research hypotheses were confirmed.

### 4.1. The Effect of Family Functioning on Adolescent Depressive Symptoms

The study findings suggest that family functioning is significantly negatively associated with adolescent depressive symptoms, indicating that adolescents with poorer family functioning experience higher levels of depressive symptoms. This result aligns with the family functioning pattern theory and is consistent with previous research [53–55]. Poor family functioning is characterized by distant or tense family relationships, low cohesion, ineffective communication among members, and infrequent and poor quality interactions, which are related to a tense and suppressive family atmosphere. In such an environment, adolescents may experience prolonged high‐stress states associated with negative emotional problems [56]. Perceived parental conflict is associated with middle school students’ positive perception of their families and the outside world, and is related to a negative coping style and reduced emotional regulation ability, which may be associated with depressive emotions [57]. Furthermore, parents who exhibit good family functioning will encourage adolescents to express their inner feelings directly from an early age. They will also pay timely attention to any emotional issues encountered during their growth process and provide appropriate solutions. Conversely, poor family functioning is related to insufficient parental involvement in children’s lives as well as neglect of attention which is associated with psychological problems. The study suggests that improving family functioning may be associated with lower levels of adolescent depressive symptoms.

However, it is important to acknowledge that this relationship may not be static across developmental stages. A longitudinal cross‐lagged study by Wang et al. [58] revealed that while family functioning at baseline significantly predicted adolescent depressive symptoms 1 year later, this predictive effect was not consistently significant at subsequent time points. Moreover, their findings suggested a potential bidirectional relationship, whereby adolescent depressive symptoms may also negatively impact family functioning over time. Despite these complexities, our cross‐sectional findings provide support for the importance of family functioning as an environmental factor associated with adolescent mental health in the Chinese cultural context, where family harmony and cohesion are particularly valued.

### 4.2. The Mediating Role of Insecure Attachment

The study revealed that insecure attachment partially mediated the relationship between family functioning and adolescent depressive symptoms. Specifically, while family functioning demonstrated a direct association with adolescent depressive symptoms, the strength of this relationship decreased after accounting for the mediating role of insecure attachment. This indicates that insecure attachment serves as a significant pathway through which family functioning is related to depressive symptoms. Furthermore, the results showed a significant positive correlation between insecure attachment and adolescent depressive symptoms, suggesting that higher levels of insecure attachment are associated with an increased likelihood of depressive symptoms. The results were consistent with previous studies [25, 26, 30].

These findings are largely consistent with previous research, although the literature reveals some important nuances. A multilevel meta‐analysis by Spruit et al. [27] which synthesized 643 effect sizes from 123 independent samples, confirmed a significant moderate association between insecure attachment and depression in children and adolescents. Notably, however, they found that cross‐sectional studies yielded larger effect sizes than longitudinal studies, suggesting that the prospective relationship between attachment and depression may be more modest than concurrent associations imply. Additionally, a meta‐analysis by Cortés‐García et al. [59] indicated that the indirect effect of insecure attachment on depression was significant primarily in adult populations, while evidence in adolescent samples was less robust. These findings suggest that attachment may be one of several mechanisms linking family environment to depression, and that other factors, such as emotion regulation strategies and cognitive vulnerabilities, may also play important mediating roles.

The quality of early life attachment to caregivers plays a crucial role in shaping adolescents’ internal working models [60], and this dysfunctional cognitive‐emotional model is associated with higher odds of depressive symptoms [61]. Individuals with high levels of insecure attachment are more susceptible to emotional problems and depressive symptoms. For adolescents, a negative family environment is associated with insecure attachment development and exhibit suspicion and anxiety in interpersonal relationships, as well as difficulty seeking social support. This may be associated with the onset of depressive symptoms. Therefore, it is necessary for family members to strive towards fostering a supportive and understanding environment. This includes making efforts to communicate emotions in a constructive manner and addressing conflicts through positive resolution strategies, rather than attempting to eliminate them entirely. Additionally, by reducing excessive control and offering consistent emotional and practical support, families can help adolescents build secure attachment styles, which may, in turn, reduce the risk of depressive symptoms.

### 4.3. The Moderating Role of SPS

The study revealed that SPS moderated the association between family functioning on adolescent depressive symptoms, as well as the mediating effect of family functioning on adolescent depressive symptoms through insecure attachment. This finding aligns with the environmental sensitivity model and contributes to empirical exploration of the model from a family perspective.

While our findings are consistent with prior studies highlighting the moderating role of SPS in the relationship between environmental factors and psychological outcomes [35–37], it is worth noting that empirical evidence in this area has been mixed. For example, Weyn et al. examined whether parenting style moderated the relationship between SPS and internalizing problems in Dutch adolescents during the COVID‐19 pandemic [62]. Although they found a significant association between SPS and internalizing problems, the hypothesized moderating effect of parenting style was not significant. Similarly, Liss et al. [63] reported that while SPS independently predicted both anxiety and depression, the interaction between SPS and negative parental environment was significant only for depression, not for anxiety. These inconsistencies may be attributed to several factors, including differences in how environmental variables are operationalized (e.g., global family functioning vs. specific parenting dimensions), cultural variations in family dynamics, and developmental differences across samples. Our study, focusing specifically on overall family functioning among Chinese adolescents, provides support for the differential susceptibility hypothesis within this particular cultural and developmental context.

Specifically, in comparison to children with lower sensitivity to the environment, those with higher sensitivity show stronger associations with both positive and negative environmental factors, such as family functioning and insecure attachment. When family functioning is well and secure attachment levels are high, highly sensitive children benefit more from these positive relationships and exhibit fewer depressive symptoms; however, when family functioning is poor and insecure attachment levels are high, highly sensitive children experience greater harm in negative relationships and display more depressive symptoms. SPS reflects individual differences in cognitive processing depth and complexity, emotional and physiological reactivity, sensitivity to subtle stimuli, and susceptibility to overstimulation [16]. Previous research have also indicated that children exhibiting “sensitivity” traits are responsive to parental nurturing [64]. In a positive nurturing environment, such as a secure parent–child attachment, highly SPS children will experience better development. Conversely, in a negative nurturing environment, such as an insecure parent–child attachment, highly SPS children will experience worse development and are more likely to encounter emotional difficulties. The results of this study and prior research demonstrate that SPS is associated with stronger relationships between poor family functioning and depressive symptoms. In an unfavorable family environment, highly SPS adolescents show stronger associations with negative family circumstances. This suggests that high SPS is associated with stronger relationships between unfavorable family environment and depressive symptoms. Therefore, for these high SPS adolescents, it is crucial for parents to create a positive family environment based on their children’s characteristics, improve family functioning, and to establish secure attachment patterns in order to prevent depressive symptoms effectively. Beyond the theoretical implications, the results underscore the need for practical, targeted interventions for highly sensitive adolescents. Approaches, such as emotion regulation training, mindfulness practices, and attachment‐focused parent–child interventions, may enhance regulatory skills, promote secure attachment, and reduce the negative impact of poor family functioning. Integrating these strategies into family or school‐based programs may offer meaningful support for adolescents with high SPS.

### 4.4. The Relationship Between Attachment and SPS

In addition, this study examined the relationship between attachment and SPS. The results indicated a significant positive correlation between insecure attachment and SPS. Specifically, individuals with higher levels of insecure attachment exhibited greater sensitivity. This finding aligns with previous literature, which has demonstrated that insecure attachment styles, such as anxious attachment, render individuals more sensitive to environmental threats, and emotional cues [65]. However, the nature and directionality of this relationship remain subjects of ongoing debate. Greven et al. [16] in their comprehensive review of environmental sensitivity, proposed that high sensitivity may actually precede and contribute to the development of attachment patterns, rather than the reverse. According to this view, highly sensitive children may be more reactive to inconsistent or unresponsive caregiving, which could increase their vulnerability to developing insecure attachment. This suggests a potential bidirectional or transactional relationship between SPS and attachment that cannot be fully captured by cross‐sectional designs.

These individuals tend to closely monitor others’ reactions to mitigate fears of rejection or neglect [66]. This heightened vigilance is consistent with the characteristics of highly sensitive individuals, particularly in terms of emotional empathy and depth of information processing [67]. Furthermore, individuals with insecure attachment often display heightened vigilance and self‐protective mechanisms when encountering emotional conflicts, potentially amplifying their sensitivity to external stimuli [68]. While this increased sensitivity may enhance adaptability to some extent, it also elevates the risk of emotional vulnerability. Given the cross‐sectional nature of our study, we cannot determine causal directionality between these constructs. Therefore, future research should explore the dynamic interplay between attachment styles and high sensitivity using longitudinal designs and develop targeted interventions to assist these individuals in regulating their emotions and adapting to their environment more effectively.

### 4.5. Limitations

This study investigates the association between family functioning and adolescent depressive symptoms, as well as the underlying mechanisms, providing a novel perspective on how SPS influences family functioning to impact adolescent depressive symptoms. However, the study has several limitations that must be addressed.

First, this is a cross‐sectional study, which inherently limits the ability to infer causal relationships or developmental trajectories. Although a longitudinal design would provide stronger evidence for causal mechanisms, practical constraints related to school schedules, limited access to participants, and resource considerations prevented the collection of multiwave data in the present study. Future research employing longitudinal designs is therefore necessary to test the causal dynamics over time. Second, the study relied on a convenience sample, which limits the generalizability of the findings. The sample may not adequately represent the broader population of adolescents, and thus the conclusions should be interpreted with caution. Future research should aim to include more representative and diverse samples. In addition, the observed urban–rural differences suggest meaningful contextual heterogeneity, indicating that future studies could benefit from multigroup or stratified sampling strategies to better capture population diversity. Third, the use of self‐reported questionnaires to assess attachment and family functioning introduces potential biases, such as social desirability or subjective perceptions that may not accurately reflect actual dynamics. Future studies should consider incorporating multimethod approaches, such as observational methods or interviews, to validate findings. Additionally, shared method variance is a concern, as all measures in the study relied on self‐reports, which could inflate observed associations. For example, adolescents with depressive symptoms may have a more negative perception of family functioning, biasing the results. Lastly, although our sample size of 503 participants meets the recommended requirements for moderated mediation analyses, a larger sample would provide greater statistical power and allow for more robust subgroup analyses.

## 5. Conclusions

In summary, this study suggests that insecure attachment mediates the relationship between family functioning and adolescent depressive symptoms, while SPS moderates the relationships between insecure attachment and adolescent depressive symptoms, as well as between family functioning and adolescent depressive symptoms. Furthermore, SPS also demonstrates a moderated mediation effect, influencing both the direct and indirect pathways between family functioning and adolescent depressive symptoms through insecure attachment. These findings have important theoretical and practical implications for understanding adolescent depressive symptoms. The study provides empirical support for the environmental sensitivity theory, showing that the impact of SPS on depressive symptoms may vary under different family stress conditions. Furthermore, the findings highlight the importance of focusing on adolescents with high levels of SPS and poor family functioning in mental health promotion efforts, which could improve prevention and intervention strategies for adolescent depressive symptoms.

## 6. Practical Implications

The findings of this study highlight the importance of family functioning in shaping adolescent mental health, particularly in relation to depressive symptoms. For adolescents, especially those with high levels of SPS, family dynamics play a critical role in either exacerbating or mitigating depressive symptoms. Based on these results, several practical implications can be drawn.

Since family functioning significantly influences adolescent depressive symptoms, interventions should prioritize improving family dynamics. Family therapy or parenting programs that focus on communication, emotional support, and conflict resolution can help foster a nurturing environment. This is particularly crucial for adolescents with high SPS, who may be more emotionally sensitive to family stressors. Given that SPS moderates the relationship between family functioning and depressive symptoms, mental health professionals should be aware of how adolescents’ heightened sensitivity to environmental stimuli may affect their emotional well‐being. Tailored interventions, such as emotional regulation training and coping skills development, can help sensitive adolescents better manage their emotional responses and reduce the impact of negative family dynamics on their mental health. Schools should consider implementing programs that support both family engagement and emotional resilience in students. Creating a school environment that reduces stress and offers emotional support can be especially beneficial for adolescents with high SPS. Training school counselors and teachers to recognize the signs of sensitivity and depressive symptoms can help in providing early intervention. Early identification of adolescents at risk due to poor family functioning or high SPS can help prevent the escalation of depressive symptoms. Screening tools for both family dysfunction and SPS traits can assist in identifying adolescents who may benefit from targeted interventions. Proactive mental health promotion programs aimed at improving family relationships and emotional resilience should be implemented in both community and school settings. By addressing both family functioning and individual sensitivities, interventions can more effectively prevent and reduce depressive symptoms in adolescents. This multifaceted approach holds the potential to support the mental well‐being of sensitive adolescents and promote healthier family environments.

## Funding

This study was supported in part by the Fundamental Research Funds for the Provincial Universities (Grant JFYQPY202302), the Heilongjiang Provincial Philosophy and Social Science Research Planning Project (Grant 21SH214), the National Natural Science Foundation of China (Grant 72204067), and the Ministry of Education Humanities and Social Sciences Foundation of China (Grants 21YJAZH046 and 25YJAZH235).

## Ethics Statement

This study was approved by the institutional review board at Harbin Medical University, Daqing (Approval Number: HMUDQ20231008001). Written informed consent was obtained from all participants and their parents.

## Consent

The authors have nothing to report.

## Conflicts of Interest

The authors declare no conflicts of interest.

## Data Availability

The data that support the findings of this study are available upon request from the corresponding author.

## Appendix A

The table includes the detailed data for the reliability and validity test conducted on the four constructs: family function (A), insecure attachment (B), sensory processing sensitivity (C), and depressive symptoms (D). It contains the estimates, standard errors, critical ratios, *p*‐values, standardized estimates, composite reliability (CR), and average variance extracted (AVE) for each item in the test.

**Table A1:** Reliability and validity test.

Variable	Item	Estimate	SE	C.R	*p*‐Value	Std estimate	CR	AVE
A	A1	1.000	—	—	—	0.739	0.924	0.502
	A2	0.984	0.062	15.916	^∗∗∗^	0.711		
	A3	0.938	0.061	15.493	^∗∗∗^	0.693		
	A4	0.996	0.063	15.905	^∗∗∗^	0.711		
	A5	1.034	0.065	15.919	^∗∗∗^	0.711		
	A6	1.043	0.063	16.544	^∗∗∗^	0.737		
	A7	1.007	0.062	16.163	^∗∗∗^	0.721		
	A8	0.802	0.055	14.522	^∗∗∗^	0.653		
	A9	0.953	0.062	15.346	^∗∗∗^	0.687		
	A10	1.044	0.064	16.332	^∗∗∗^	0.728		
	A11	0.989	0.063	15.659	^∗∗∗^	0.700		
	A12	1.014	0.064	15.860	^∗∗∗^	0.709		

B	B1	1.000	—	—	—	0.684	0.912	0.537
	B2	1.098	0.073	15.102	^∗∗∗^	0.737		
	B3	1.119	0.076	14.781	^∗∗∗^	0.720		
	B4	1.134	0.073	15.533	^∗∗∗^	0.760		
	B5	1.128	0.075	15.066	^∗∗∗^	0.735		
	B6	1.136	0.071	16.089	^∗∗∗^	0.791		
	B7	1.084	0.075	14.456	^∗∗∗^	0.703		
	B8	1.104	0.076	14.572	^∗∗∗^	0.709		
	B9	1.051	0.069	15.300	^∗∗∗^	0.748		

C	C1	1.000	—	—	—	0.775	0.923	0.571
	C2	1.054	0.060	17.555	^∗∗∗^	0.743		
	C3	0.922	0.066	14.037	^∗∗∗^	0.612		
	C4	1.080	0.057	18.839	^∗∗∗^	0.787		
	C5	1.076	0.058	18.543	^∗∗∗^	0.777		
	C6	1.067	0.057	18.559	^∗∗∗^	0.778		
	C7	1.076	0.057	18.780	^∗∗∗^	0.785		
	C8	1.073	0.058	18.399	^∗∗∗^	0.772		
	C9	1.024	0.057	17.875	^∗∗∗^	0.754		

D	D1	1.000	—	—	—	0.795	0.974	0.653
	D2	1.026	0.049	20.977	^∗∗∗^	0.809		
	D3	1.036	0.049	21.300	^∗∗∗^	0.818		
	D4	1.025	0.049	21.032	^∗∗∗^	0.810		
	D5	1.047	0.051	20.608	^∗∗∗^	0.799		
	D6	1.012	0.049	20.465	^∗∗∗^	0.794		
	D7	0.992	0.048	20.594	^∗∗∗^	0.798		
	D8	0.978	0.047	20.741	^∗∗∗^	0.802		
	D9	1.028	0.047	21.876	^∗∗∗^	0.834		
	D10	1.002	0.048	21.067	^∗∗∗^	0.811		
	D11	1.037	0.046	22.676	^∗∗∗^	0.855		
	D12	0.961	0.048	19.945	^∗∗∗^	0.779		
	D13	0.999	0.050	20.054	^∗∗∗^	0.783		
	D14	1.050	0.047	22.363	^∗∗∗^	0.847		
	D15	1.036	0.049	21.312	^∗∗∗^	0.818		
	D16	0.960	0.051	18.804	^∗∗∗^	0.745		
	D17	0.998	0.048	20.743	^∗∗∗^	0.802		
	D18	0.985	0.048	20.459	^∗∗∗^	0.794		
	D19	1.042	0.049	21.196	^∗∗∗^	0.815		
	D20	1.073	0.048	22.204	^∗∗∗^	0.843		

*Note:* A, family function; B, insecure attachment; C, sensory processing sensitivity; D, depressive symptom.

^∗∗∗^ *p* < 0.01.
